# Predicting the future of ALS: the impact of demographic change and potential new treatments on the prevalence of ALS in the United Kingdom, 2020–2116

**DOI:** 10.1080/21678421.2019.1587629

**Published:** 2019-04-09

**Authors:** Alison Gowland, Sarah Opie-Martin, Kirsten M. Scott, Ashley R. Jones, Puja R. Mehta, Christine J. Batts, Cathy M. Ellis, P. Nigel Leigh, Christopher E. Shaw, Jemeen Sreedharan, Ammar Al-Chalabi

**Affiliations:** 1 Department of Basic and Clinical Neuroscience, King’s College London, Maurice Wohl Clinical Neuroscience Institute, London, UK,; 2 Department of Clinical Neuroscience, University of Cambridge, Cambridge, UK,; 3 King’s College Hospital, London, UK,; 4 Kent and Canterbury Hospital, East Kent Hospital NHS University Foundation Trust, Canterbury, UK,; 5 Department of Neuroscience, Brighton and Sussex Medical School, Trafford Centre for Biomedical Research, University of Sussex, Brighton, UK, and; 6 United Kingdom Dementia Research Institute, Maurice Wohl Clinical Neuroscience Institute, Institute of Psychiatry, Psychology and Neuroscience, King’s College London, London, UK

**Keywords:** Epidemiology, models, survival, *SOD1*, therapy, genetics

## Abstract

*Objective:* To model the effects of demographic change under various scenarios of possible future treatment developments in ALS. *Methods:* Patients diagnosed with ALS at the King’s College Hospital Motor Nerve Clinic between 2004 and 2017, and living within the London boroughs of Lambeth, Southwark, and Lewisham (LSL), were included as incident cases. We also ascertained incident cases from the Canterbury region over the same period. Future incidence of ALS was estimated by applying the calculated age- and sex-specific incidence rates to the UK population projections from 2020 to 2116. The number of prevalent cases for each future year was estimated based on an established method. Assuming constant incidence, we modelled four possible future prevalence scenarios by altering the median disease duration for varying subsets of the population, to represent the impact of new treatments. *Results:* The total number of people newly diagnosed with ALS per year in the UK is projected to rise from a baseline of 1415 UK cases in 2010 to 1701 in 2020 and 2635 in 2116. Overall prevalence of ALS was predicted to increase from 8.58 per 100,000 persons in 2020 to 9.67 per 100,000 persons in 2116. Halting disease progression in patients with *C9orf72* mutations would yield the greatest impact of the modelled treatment scenarios, increasing prevalence in the year 2066 from a baseline of 9.50 per 100,000 persons to 15.68 per 100,000 persons. *Conclusions:* Future developments in treatment would combine with the effects of demographic change to result in more people living longer with ALS.

## Introduction

Amyotrophic lateral sclerosis (ALS) is a rapidly progressive neurodegenerative disease of motor neurons leading to wasting, paralysis, and eventual death from respiratory failure within 3 to 5 years. The annual incidence is approximately 2 per 100,000 person-years in European (1) and US populations (2). These incidence rates have remained relatively stable over time (3). The lifetime risk is as high as 1 in 300 (4) with disease burden increasing with age.

Demographic change poses challenges for care provision and resourcing. Across developed and developing countries, the proportion of people in older age groups is predicted to increase significantly over the coming decades (5), secondary to decreasing mortality and declining fertility. The number of people aged 60 and over is expected to double globally from 841 million in 2013 to more than 2 billion in 2050, with a projected 69% global increase in the number of ALS cases worldwide by 2040 (6).

A counterpoint to the challenge of future population change is the hope offered by the possible development of new ALS treatments to slow progression and prolong survival for some or all patients. While there is not yet such a treatment, significant survival improvement might be expected in coming decades. For example, randomized controlled trial evidence shows that Riluzole can improve 12-month survival by about 35% (7), and lithium may improve the survival of a genetic subgroup (8). Edaravone, though not licensed in the UK, has been shown to slow functional decline in ALS for a subgroup of patients (9). Targeted gene therapy approaches are in development (10), and future treatments may slow or halt the progress of disease, resulting in a larger prevalent cohort of people with ALS.

Population change and its interaction with the effects of treatments have major implications for a healthcare and societal system with limited resourcing and many competing demands. We therefore modelled the effects of demographic changes under various scenarios of treatment outcomes in ALS.

## Materials and methods

### Incidence estimates

Patients diagnosed with ALS at the King’s College Hospital Motor Nerve Clinic between 1 January 2004 and 31 December 2017, and living within the London boroughs of Lambeth, Southwark, and Lewisham (LSL), were included as incident cases. These three south London boroughs were selected because they comprise the primary catchment for the centre, therefore ensuring good case ascertainment. Patients with all subtypes of ALS and all El Escorial diagnostic certainty categories (as assigned at first presentation to the clinic) were included. For patient confidentiality, postcode data was only available to the level of postcode sector (a geographical area containing approximately 3000 households), which cuts across borough boundaries, and so incident cases were weighted according to the proportion of the patient’s postcode sector that fell within one of the three relevant boroughs.

Patients were grouped by sex and age according to age at diagnosis in 5-year age bands from 15 years upwards, with an open-ended cohort for those aged 90 years and above. In the event of no cases within any particular category, we substituted 0.5 to allow extrapolation to future projections. Crude age- and sex-specific incidence rates were calculated using age- and sex-specific mid-2010 population estimates for the relevant boroughs as the denominator from Office of National Statistics (ONS) census data. Confidence intervals for crude rates were calculated using the exact method for Poisson intervals (11). These rates were standardized to the UK population structure (mid-2010 estimate) (12) using the direct standardization method. Confidence intervals for the overall age- and sex-adjusted incidence rates were calculated at the 95% level using an approximation of the standard error for a binomial proportion (13).

To provide a comparison and to increase our total sample size, incident cases from the Canterbury region arising over the same period (2004–2017) were ascertained from the SEALS register (14) which captures case referrals to the region’s motor neuron disease specialist nursing service. Because this service accepts referrals by postcode criteria, this sample was defined by postcode sector rather than by borough. Crude age- and sex-specific incidence rates were calculated using age- and sex-specific mid-2011 population data for the relevant postcode sectors as the denominator (15). Denominator estimates from 2011 were used (rather than 2010 as for the London data) because this was a UK census year for which postcode-level (rather than borough level) statistics were available. Age- and sex-specific incidence rates were standardized to the UK population structure (mid-2011 population estimate) (16) using the direct standardization method. Otherwise, the sample data were processed and analyzed in the same way as the London sample. To compare the two regional age-standardized rates, the standardized rate ratio (17) was calculated. Data from the London boroughs and the Canterbury region were pooled to provide incidence estimates from a larger sample size incorporating urban, suburban, and rural populations.

### Prevalence estimates

Point prevalence was estimated at 30 June 2010 for the London boroughs, and 27 March 2011 for the Canterbury region, based on date of availability of population estimates for the different samples. Survival data were collected from hospital and national case records. Prevalent cases were included if diagnosis occurred any time after 1 January 1994. Confidence intervals (95%) for point prevalence estimates were calculated by Wilson’s method for calculating confidence intervals for a proportion using OpenEpi software (Emory University, Atlanta, GA, USA) (18).

### Future projections

The effects of future population change on the number of incident cases of ALS were estimated by applying the calculated age- and sex-specific incidence rates generated from the pooled dataset to the main UK population projection (19) for each single year from 2020 to 2116, with population counts for each single year of age summed to correspond to our age cohorts. This generated an estimate of the number of incident cases per future year, by sex and age cohort.

### Prevalence modelling

To estimate the number of prevalent cases in future years, we used an established method (6). The number of cases per year was estimated based on the product of calculated age- and sex-specific incidence rates, the age- and sex-specific future population estimates taken from the main UK population projection described above, and the median disease duration. To generate estimates of median disease duration we performed a Kaplan Meier survival analysis using data from the whole of the SEALS Register (14). Cases were censored at death or at last recorded follow up date, with median survival (in months) calculated from date of symptom onset to the censor/event date. Cases were grouped into three age cohorts—under 40 years, 41–79 years and 80 years and over—based on findings of a previous review of age as a prognostic factor in ALS (20).

Assuming constant incidence, we modelled four different future prevalence scenarios to reflect possible developments in treatment.

1. A new Riluzole-equivalent drug that increases median survival in all affected people by 3 months.
2. A new gene therapy for *SOD1* gene mutation that increases median survival in carriers by 50%. Based on estimates of 10% of people with ALS having an ascertained family history, 20% of which can be attributed to *SOD1* mutation, and 2% of sporadic cases being *SOD1* mutation carriers (21), 4% of the total population was selected for this survival benefit.
3. A new gene therapy for *C9orf72* gene mutation that increases median survival in carriers by 50%. Ten percent (10%) of the total population was selected for this survival benefit, based on estimated overall frequency of *C9orf72* mutations in studies of familial and sporadic ALS cases (22–24).
4. A new gene therapy for *C9orf72* gene mutation that halts progression of disease. Ten percent (10%) of the total population was selected for this survival benefit, and survival for this subgroup was derived from average period life expectancies published in ONS life tables, up to the year 2066 (25).

## Results

### Incidence estimates: Lambeth, Southwark, and Lewisham (LSL) data

**Figure 1:**
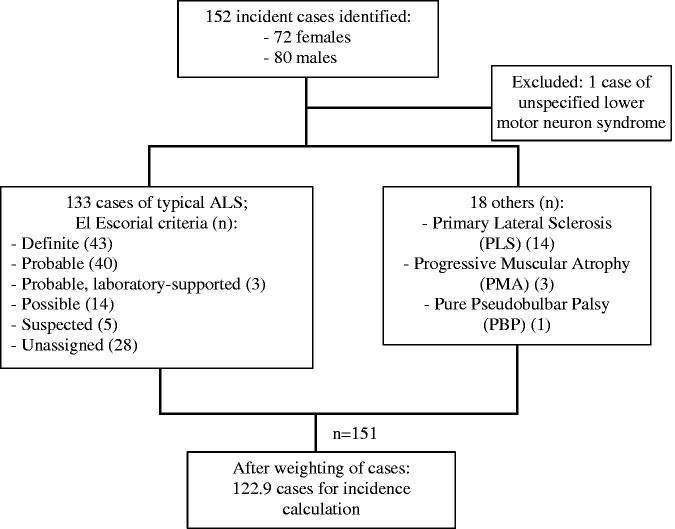
Flowchart of incidence sample characteristics: Lambeth, Southwark, and Lewisham (LSL) data.

There were 152 incident cases identified over the relevant period. Of these, 72 (47.4%) were female. One person with an unspecified lower motor neuron syndrome was excluded. There were 133 people diagnosed with typical ALS. Of these, 43 were recorded as “Definite” ALS according to El Escorial criteria, 40 “Probable”, 3 “Probable, laboratory-supported”, and 14 “Possible”. Five people with ALS classified under the now defunct category of “Suspected” were included. There were no data on El Escorial category in 28 cases. There were 14 people with primary lateral sclerosis (PLS), 3 with progressive muscular atrophy (PMA) and 1 with a pure pseudobulbar palsy, all of whom were included. After exclusions, and including partial counts as described above, there were 122.9 cases for the incidence calculation. Figure 1 illustrates the incidence sample characteristics.

The overall crude incidence rate was 1.29 per 100,000 (95% CI 1.07–1.53). The overall incidence rate standardized to the 2010 UK population was 2.10 per 100,000 (95% CI 1.97–2.22). The age-standardized rate in males was 2.27 per 100,000 (95% CI 2.08–2.46) and in females 1.93 per 100,000 (95% CI 1.76–2.10). Age- and sex-specific incidence rates are provided in the supplementary material (Supplementary Table A).

### Incidence estimates: Canterbury data

**Figure 2:**
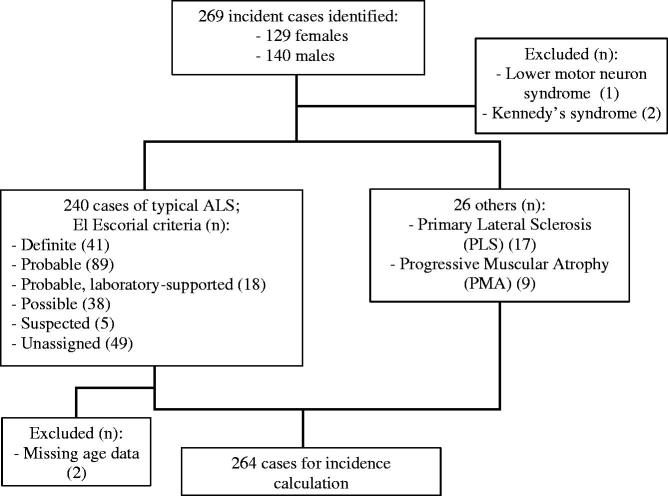
Flowchart of incidence sample characteristics: Canterbury data.

There were 269 incident cases identified from the register over the relevant period. One person with a lower motor neuron syndrome was excluded. Two people with Kennedy’s disease were excluded. Of the remaining 266, 128 (48.1%) were female. ALS was diagnosed in 240 people, and there were 17 people with PLS and 9 with PMA. Of the 240 people with typical ALS, 41 were recorded as “Definite” according to El Escorial criteria, 89 “Probable”, 18 “Probable, laboratory-supported”, and 38 “Possible”. Five people were classified under the now defunct category of “Suspected”. There were no data on El Escorial category in 49. Two were excluded due to missing age data, leaving 264 in the final incidence calculation. Figure 2 illustrates the incidence sample characteristics.

The overall crude incidence rate was 3.61 per 100,000 person-years (95% CI 3.19–4.07). The overall incidence rate standardized to the 2011 UK population was 3.25 per 100,000 person-years (95% CI 3.09–3.40). The age-standardized rate was 3.51 per 100,000 person-years for males (95% CI 2.38–3.74) and 3.01 per 100,000 person-years for females (95% CI 2.80–3.21). Age- and sex-specific incidence rates are provided in the supplementary material (Supplementary Table B). The standardized rate ratio of the Canterbury and LSL overall age-standardized rates was 1.55 (95% CI 1.43–1.67).

### Pooled incidence data

When the data for the two regions were pooled, the overall crude incidence rate was 2.27 per 100,000 person-years (95% CI 2.04–2.50). The overall incidence rate standardized to the 2010 UK population was 2.74 per 100,000 person-years (95% CI 2.59–2.88). The age-standardized rate in males was 2.96 per 100,000 person-years (95% CI 2.74–3.17) and in females 2.53 per 100,000 person-years (95% CI 2.34–2.72). Age- and sex-specific incidence rates are provided in the supplementary material (Supplementary Table C). Comparative rates are summarized in Table 1.

**Table 1:** Summary of crude and adjusted incidence rates for Lambeth, Southwark, and Lewisham (LSLS) data, Canterbury region data, and pooled data, 2004–2017.

	LSL data	Canterbury data	Pooled data
Crude incidence rate (per 100,000 per year) (95% CI)	1.29 (1.07–1.53)	3.61 (3.19–4.07)	2.27 (2.04–2.50)
Male age-adjusted incidence rate	2.27 (2.08–2.46)	3.51 (2.38–3.74)	2.96 (2.74–3.17)
Female age-adjusted incidence rate	1.93 (1.76–2.10)	3.01 (2.80–3.21)	2.53 (2.34–2.72)
Overall age- and gender-adjusted incidence rate	2.10 (1.97–2.22)	3.25 (3.09–3.40)	2.74 (2.59–2.88)
Number of cases	1084	1695	1415

### Prevalence estimates

Using the LSL dataset, there were 37 people identified as living with ALS at the relevant date. The point prevalence estimate was 5.29 per 100,000 persons (95% CI 3.84–7.30). Using the Canterbury dataset, there were 44 people identified as living with ALS at the relevant date. The point prevalence estimate was 8.30 per 100,000 persons (95% CI 6.19–11.15).

### Future projections

**Figure 3:**
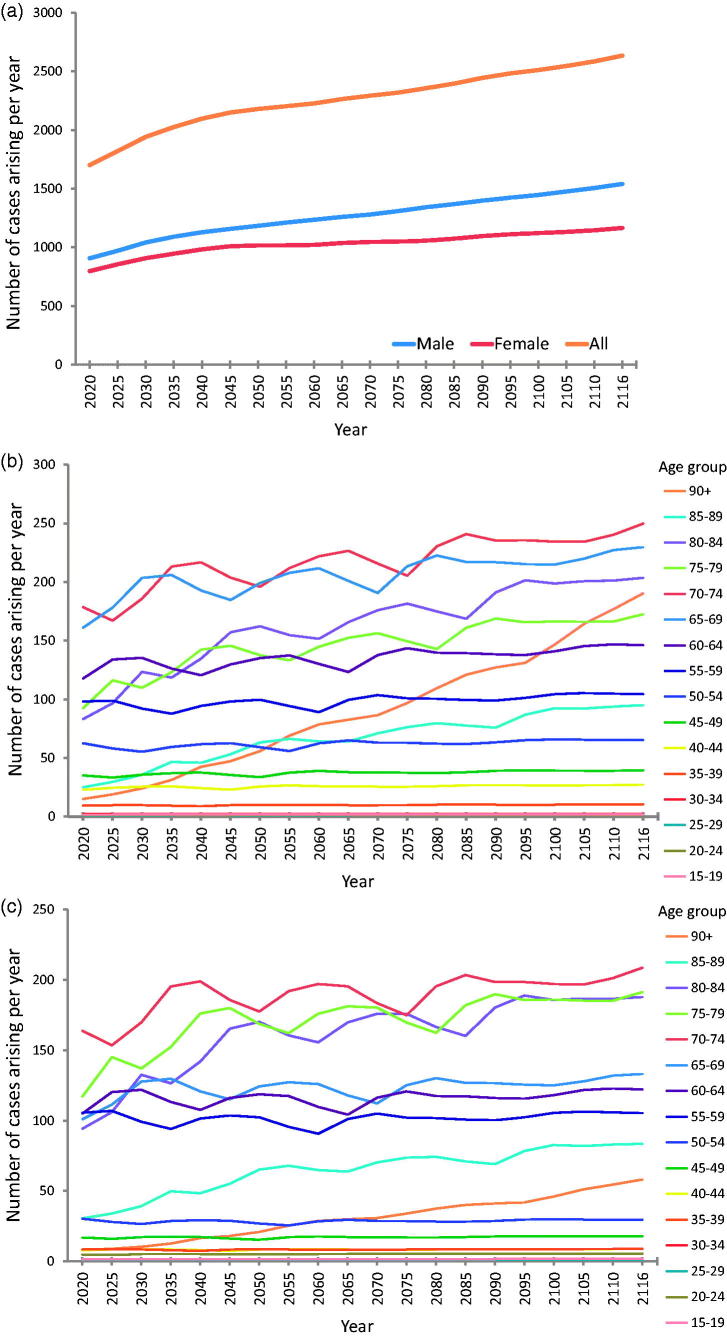
Predicted number of new cases of ALS arising in UK per year, by sex and by age group for each sex, 2020–2116. This is the illustration of future incidence projections. (a) Number of new UK cases arising per future year, by sex. Male incidence remains higher than female incidence, with a slight increase in the number of male cases arising in later years due to projected improvements in male life expectancy. (b) Number of new UK male cases arising per future year, by age cohort. Note the particularly steep rise in annual incidence rate for males in the 90+ age cohort; this is attributable to projected improvements in life expectancy for males resulting in increasingly large numbers of males reaching this age cohort, combined with a relatively high age-specific incidence rate in our sample (7.56 male cases per 100,000 persons compared to 1.99 female cases per 100,000 persons in this age cohort). (c) Number of new UK female cases arising per future year, by age cohort. This suggests an increase in the number of cases arising per year in future, particularly among the older age cohorts.

Figure 3 illustrates the projected trend of new cases arising per year, by sex and by age group for each sex. The total number of people newly diagnosed with ALS per year in the UK is projected to rise over the coming decades, from a baseline of 1415 UK cases in 2010 to 1701 in 2020 and 2635 in 2116. For males, the projected increase in new cases was from 743 in 2010 to 1540 in 2116, with population increases in the highest age cohort (over 90 years) contributing significantly to this rise. For females, the projected increase was from 672 new cases in 2010 to 1165 new cases in 2116.

### Prevalence modelling—survival analysis

A total of 953 cases were identified in the SEALS register, 434 of whom (45.4%) were female. Diagnoses other than ALS were excluded (three people with Kennedy’s syndrome, one with multiple sclerosis, one with a lower motor neuron syndrome, and one other). There were 803 cases of ALS with defined phenotypes (e.g. flail arm, flail leg, with FTD, and bulbar palsy); 50 people where no specific ALS phenotype was listed were also included. Two hundred had El Escorial “Definite” ALS, 355 “Probable”, 43 “Probable, laboratory-supported”, and 107 “Possible”. Twenty were classified under the now defunct El Escorial category of “Suspected”. There were no data on El Escorial category in 128.

**Figure 4:**
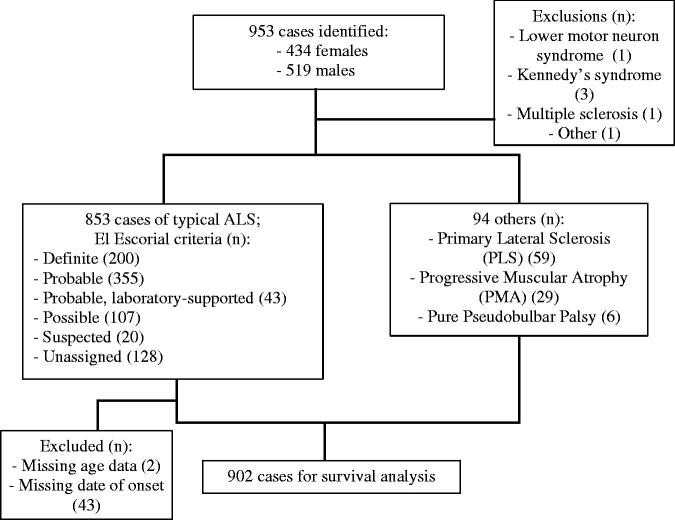
Flowchart of survival analysis sample characteristics.

There were 59 people with PLS, 29 with PMA, and 6 with a pure pseudobulbar palsy, all of whom were included. Those without a date of onset were excluded (*n* = 43), as well as those with no age at diagnosis (*n* = 2), leaving 902 for survival analysis. Figure 4 illustrates the incidence sample characteristics. Median survival estimates are displayed in Table 2.

**Table 2:** Results of Kaplan-Meier survival analysis: mean and median survival estimates (months) by age cohort.

Means and medians for survival time								
Overall								
Age cohort	Mean				Median			
	Estimate	Std. error	95% Confidence interval		Estimate	Std. error	95% Confidence interval	
			Lower bound	Upper bound			Lower bound	Upper bound
<40	67.61	10.13	47.75	87.47	56.00	26.01	5.01	106.99
40–79	63.09	4.59	54.09	72.08	35.00	1.37	32.32	37.68
80+	42.82	7.38	28.35	57.29	26.00	3.31	19.51	32.49
Overall	61.46	3.94	53.73	69.18	34.00	1.06	31.92	36.08

At baseline, using the pooled incidence rates and the above survival estimates, overall prevalence of ALS was predicted to increase from 8.58 per 100,000 persons in 2020 to 9.67 per 100,000 persons in 2116. Prevalence estimates resulting from other scenarios are displayed in Table 3 and illustrated individually in comparison to the baseline scenario in Figure 5.

Figure 5Future prevalence estimates for each modelled scenario compared to baseline. (a) Baseline compared to a treatment that prolongs survival in all cases by three months. (b) Baseline compared to a treatment that prolongs median survival by 50% in the subgroup of patients with *SOD1* mutation. (c) Baseline compared to a treatment that prolongs median survival by 50% in the subgroup of patients with *C9orf72* mutation. (d) Baseline compared to a treatment that halts disease progression in the subgroup of patients with *C9orf72* mutation, so that the life expectancy of these patients reverts to that predicted for their age, sex, and year cohort. This scenario generates the most significant difference in disease prevalence compared to baseline. Note that the range of the x-axis is different for (d) (2020–2066) compared to (a–c) (2020–2116) due to more limited availability of projected life expectancy data. Note that for all parts of Figure 5, the y-axis lower limit is eight (prevalent cases per 100,000 persons), not 0, for better illustration of the range of prevalence estimates.

**Figure:**
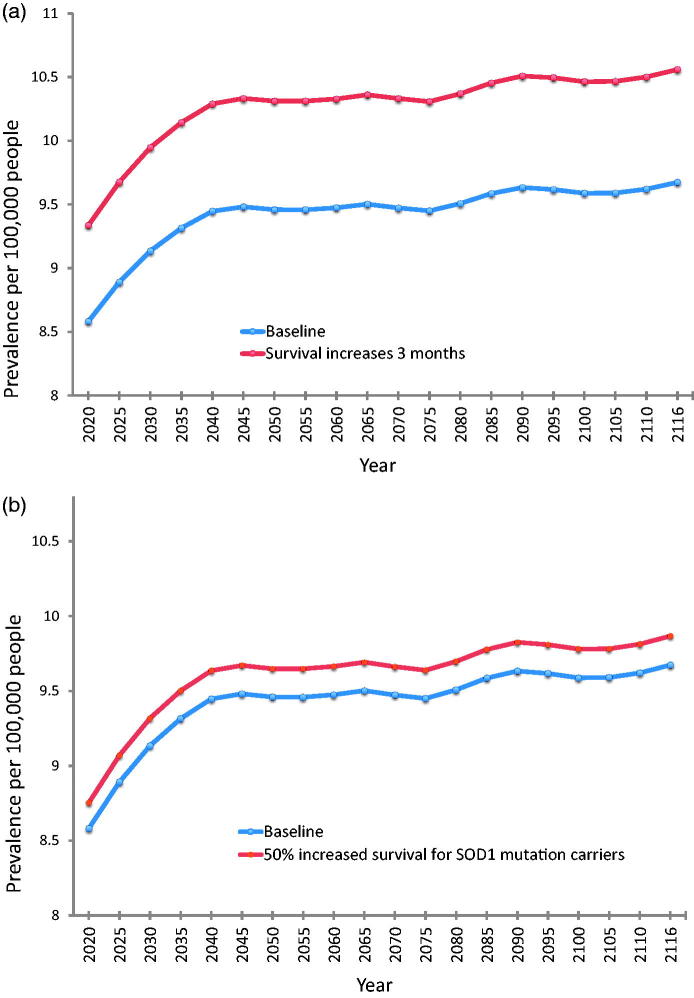

**Figure:**
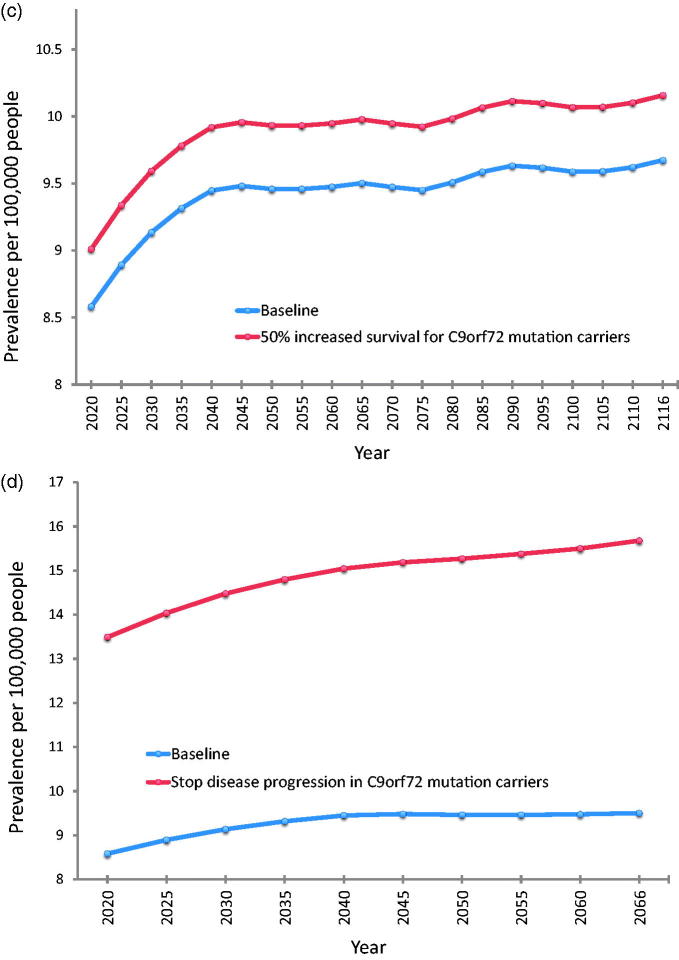

**Table 3:** Summary of prevalence estimates for males, females, and both at baseline and for each modeled scenario, showing first and last year of available data.

	Male prevalence per 100,000		Female prevalence per 100,000		Overall prevalence per 100,000	
	2020	2116	2020	2116	2020	2116
Baseline	9.34	11.12	7.88	8.65	8.58	9.67
Scenario 1 (overall 3-month survival increase)	10.16	12.15	8.57	9.44	9.34	10.56
Scenario 2 (50% survival increase in *SOD1* mutation carriers)	9.52	11.34	8.04	8.82	8.75	9.87
Scenario 3 (50% survival increase in *C9orf72* mutation carriers)	9.80	11.68	8.27	9.08	9.01	10.16
	2020	2066	2020	2066	2020	2066
Baseline	9.34	10.55	7.88	8.67	8.58	9.50
Scenario 4 (halt progression in *C9orf72* mutation carriers)^a^	14.55	17.24	12.44	14.36	13.49	15.68

a Scenario 4 comparisons are shown for the years 2020 and 2066, which is the last year for which projected life expectancy data is available.

## Discussion

Our study pooled data from two regions of south-east England to generate robust incidence rate estimates for ALS in the UK, and used these to predict the effects of future demographic and scientific change on the numbers of people living with ALS in the UK up to the year 2116. Demographic change will almost double the number of people newly diagnosed with ALS each year, from a baseline of 1415 cases in 2010 to 2635 cases in 2116. In combination with this, future developments in treatment for ALS can be predicted to alter the prevalence of the disease by prolonging survival to varying degrees. Halting disease progression entirely in patients who are *C9orf72* mutation carriers would yield the greatest impact of the scenarios we modelled, increasing prevalence in 2066 from a predicted baseline of 9.50 per 100,000 persons to 15.68 per 100,000 persons. The discovery of a new drug treatment equivalent to the only currently licensed drug therapy in the UK (Riluzole) would generate the next greatest impact, with an estimated ALS prevalence of 10.56 per 100,000 in 2116 compared to a baseline of 9.56 per 100,000.

This study represents an updating of previously published ALS epidemiological research, including data from the same regions over different study periods. For example, an earlier study from our research group (4) presented incidence data from the LSL boroughs from 1997 to 2004, and reported a crude incidence rate of 1.20 cases per 100,000 person-years, which is similar to our crude incidence rate of 1.29 cases per 100,000 person-years. It was adjusted to the England and Wales population, rather than the whole of the UK, so comparisons of overall age- and sex-adjusted rates are not possible. Using current incidence estimates to make future ALS prevalence predictions is a technique deployed previously (6) to make global predictions for the year 2040. In relation to Europe, the earlier study predicted that the number of individuals living with ALS would increase by 20% between the years 2015 and 2040. Our model predicts a 30% increase in UK cases over approximately the same period (2016–2040), based on more detailed survival estimates (by age group and by sex), as well as locally derived age- and sex-specific incidence rates. The average direct medical cost for someone with ALS is estimated at US$3436 per month (26). The increase in prevalence over our whole study period (2020–2116) would therefore be predicted to add at least an additional US$95m to current healthcare costs in the UK. Our study goes further in attempting to model the impact of possible (and plausible) future developments in treatment, all of which would combine with the effect of population increase to result in more people living longer with ALS. This effect is likely to be generalizable to other neurodegenerative diseases, which are also predicted to become more prevalent in an aging population e.g. Alzheimer’s dementia (27).

Our study also found significantly different incident rates between two parts of south-east England, an inner city area (LSL) and a more mixed suburban/rural region (Canterbury), with higher rates estimated from the Canterbury region data. This is likely to derive in part from the different demographic structures of the two areas, with 21% of the Canterbury population aged over 65, compared to just 9% for the LSL population. Our inner city estimate is nonetheless higher than a previous estimate using the same sample area (4), which reported an overall adjusted incidence rate of 1.66—though this was standardized to an England and Wales population rather than to the whole UK as in our study. The pooled data yielded a crude incidence rate (2.27 per 100,000) comparable to that quoted in European register studies (e.g. 2.16 per 100,000 in (1)), with our slightly higher rate possibly explained by our inclusion of a lower motor neuron predominant and upper motor neuron predominant forms of ALS rather than only typical ALS. This broader focus is likely a contributing factor to our higher point prevalence estimates compared to European median estimates (28), as we include subtypes like PLS that tend to have considerably longer median survival times than ALS (29). It could be argued that our decision to include these upper or lower motor neuron predominant forms, albeit in their relatively small absolute case numbers, dilutes the ALS epidemiological dataset and limits comparability with other studies. However, this enables us to better capture cases where there may be some doubt about the precise motor neuron disease diagnosis (30), as well as better reflect the likely future demands on motor nerve clinic services overall.

Similarly, current treatments under exploration tend to focus on potential targets within the known pathophysiological models of ALS (10) therefore our higher prevalence estimates under the different treatment scenarios (compared to baseline) may be overestimates, given that we have included non-typical ALS cases in the baseline incidence calculations. We have made some assumptions about the different treatment scenarios, which were selected based on biological plausibility and some early findings from animal models; for example, a trial of antisense oligonucleotide therapy in transgenic (*SOD1*-G93A) rats and resulted in reduced mutated *SOD1* as well as extended survival (31); our selection of 50% survival extensions in two of our modelled scenarios remains somewhat arbitrary, and could be refined in further studies to reflect ongoing work in animal models and results from early human clinical trials.

This study’s findings may also be limited by the assumptions we have made about population changes. We used the Office for National Statistics main population projection model, which makes certain assumptions about aging, migration and other factors. There is a developing body of evidence to suggest that the incidence and prevalence of ALS may vary with ethnicity and ancestry (32), therefore it is plausible that future changes in ethnic diversity in the UK could affect our projections. Alternative ONS projections that assume, for example, higher levels of inward migration, could be used to repeat the analyses undertaken in this study. It is also possible that there are environmental modifiers of ALS epidemiology that are not considered in our study, such as birth cohort effects restricted to particular groups (33).

We did not model any scenarios wherein disease onset could be prevented altogether within any particular sub-group of ALS patients; this could provide an interesting comparison to the scenarios modelled here, all of which predict increased future disease prevalence, but is currently unlikely given the frequency of ALS in the population and the consequent statistical challenge in developing an effective screening test. Another recommendation for further work in this field is to continue the collection and refinement of robust epidemiological data about ALS, which should be promoted by the emergence of a national ALS patient register as well as use of capture–recapture techniques to improve the reliability of incidence and prevalence estimates.

## Supplementary Material

Supplementary_files.docx
