# Supplementary material for: Predicting the future of ALS: the impact of demographic change and potential new treatments on the prevalence of ALS in the United Kingdom, 2020–2116
Source: Amyotroph Lateral Scler Frontotemporal Degener. 2019 Apr 9;20(3-4):264–74. doi: 10.1080/21678421.2019.1587629 (PMC6567553; doi:10.1080/21678421.2019.1587629)
Supplement: Supplementary_files.docx [file IAFD_A_1587629_SM7512.docx]

**Supplementary material**

Supplementary Table A. Age- and sex-specific ALS incidence rates for Lambeth, Southwark and Lewisham, 2004 – 2017.

|  | **Incidence rates (cases arising per 100,000 per year)** | | |
| --- | --- | --- | --- |
| **Age cohort** | **Male** | **Female** | **All** |
| 15-19 | 0.16 | 0.15 | 0.16 |
| 20-24 | 0.10 | 0.19 | 0.15 |
| 25-29 | 0.07 | 0.07 | 0.07 |
| 30-34 | 0.13 | 0.08 | 0.10 |
| 35-39 | 0.61 | 0.19 | 0.41 |
| 40-44 | 0.61 | 0.43 | 0.52 |
| 45-49 | 1.70 | 0.43 | 1.06 |
| 50-54 | 1.70 | 1.52 | 1.61 |
| 55-59 | 3.31 | 2.75 | 3.03 |
| 60-64 | 4.67 | 3.77 | 4.21 |
| 65-69 | 9.82 | 4.64 | 7.08 |
| 70-74 | 8.60 | 5.83 | 7.11 |
| 75-79 | 5.82 | 7.10 | 6.53 |
| 80-84 | 0.70 | 5.96 | 3.88 |
| 85-89 | 3.60 | 7.05 | 5.92 |
| 90+ | 7.11 | 1.65 | 3.38 |

Supplementary Table B. Age- and sex-specific ALS incidence rates for Canterbury region, 2004-2017.

|  | **Incidence rates (cases arising per 100,000 per year)** | | |
| --- | --- | --- | --- |
| **Age cohort** | **Male** | **Female** | **All** |
| 15-19 | 0.16 | 0.17 | 0.16 |
| 20-24 | 0.17 | 0.33 | 0.25 |
| 25-29 | 0.21 | 0.20 | 0.20 |
| 30-34 | 0.22 | 0.21 | 0.22 |
| 35-39 | 0.20 | 0.75 | 0.49 |
| 40-44 | 1.30 | 0.31 | 0.80 |
| 45-49 | 1.58 | 1.23 | 1.40 |
| 50-54 | 4.28 | 0.69 | 2.46 |
| 55-59 | 5.41 | 5.82 | 5.62 |
| 60-64 | 6.62 | 5.98 | 6.29 |
| 65-69 | 9.09 | 6.49 | 7.74 |
| 70-74 | 12.60 | 11.09 | 11.81 |
| 75-79 | 9.66 | 10.81 | 10.29 |
| 80-84 | 15.38 | 11.76 | 13.23 |
| 85-89 | 6.89 | 2.74 | 4.17 |
| 90+ | 7.80 | 2.85 | 4.18 |

Supplementary Table C. Age- and sex-specific ALS incidence rates for pooled data from Lambeth, Southwark and Lewisham, and Canterbury region, 2004-2017.

|  | **Incidence rates (cases arising per 100,000 per year)** | | |
| --- | --- | --- | --- |
| **Age cohort** | **Male** | **Female** | **All** |
| 15-19 | 0.08 | 0.08 | 0.08 |
| 20-24 | 0.06 | 0.24 | 0.16 |
| 25-29 | 0.05 | 0.05 | 0.05 |
| 30-34 | 0.10 | 0.06 | 0.08 |
| 35-39 | 0.43 | 0.38 | 0.40 |
| 40-44 | 0.87 | 0.38 | 0.63 |
| 45-49 | 1.65 | 0.78 | 1.21 |
| 50-54 | 2.92 | 1.13 | 2.01 |
| 55-59 | 4.43 | 4.41 | 4.42 |
| 60-64 | 5.87 | 5.14 | 5.50 |
| 65-69 | 9.35 | 5.82 | 7.50 |
| 70-74 | 11.08 | 9.02 | 9.99 |
| 75-79 | 8.23 | 9.39 | 8.87 |
| 80-84 | 10.17 | 9.65 | 9.86 |
| 85-89 | 5.83 | 4.21 | 4.76 |
| 90+ | 7.56 | 1.99 | 3.57 |
